# 2549 Characterizing the expression kinetics of HIV-1 envelope protein

**DOI:** 10.1017/cts.2018.54

**Published:** 2018-11-21

**Authors:** Djin-Ye Oh, Lihong Liu, Benjamin Trinité, En-Wei Hu-Van Wright, Vincent Sahi, Yaoxing Huang, David N. Levy, David D. Ho

**Affiliations:** Rockefeller University

## Abstract

OBJECTIVES/SPECIFIC AIMS: Characterize the expression kinetics of HIV-1 Envelope and their relationship to virus production at the cellular level. METHODS/STUDY POPULATION: In vitro and ex vivo laboratory analyses. RESULTS/ANTICIPATED RESULTS: Initial studies addressing the kinetics of cell surface. Envelope (Env) expression reveal that Env expression to peaks on day 2 post infection. Next steps include a series of experiments to compare the kinetics of Env cell surface expression with broadly neutralizing antibody (bNAb)-mediated ADCC and the characterization of virus production kinetics in this same context. To be maximally effective, ADCC elimination of infected cells should occur before peak Env expression. DISCUSSION/SIGNIFICANCE OF IMPACT: Potent bNAbs to HIV-1 recognize vulnerable sites on the HIV-1 Envelope (Env) protein and are of great clinical interest due to their potential use in the prevention and treatment of HIV-1 infection. Their effectiveness depends not only on the neutralization of viral infectivity, but also on the elimination of productively infected cells via antibody-dependent cellular cytotoxicity (ADCC). On a cellular level, ADCC dynamics are determined by the timing and level of Env expression on the surface of HIV-infected cells. This study aims to delineate the expression kinetics of HIV-1 Envelope and their relationship to virus production. We expect that it will provide new insights into the utility of bNAb-mediated ADCC in treating and possibly curing HIV-1 infection; therefore results might have substantial impact on future HIV treatment strategies.

